# Ki-67 response-guided preoperative chemotherapy for HER2-positive breast cancer: results of a randomised Phase 2 study

**DOI:** 10.1038/s41416-020-0815-9

**Published:** 2020-04-02

**Authors:** Hirofumi Mukai, Takeshi Yamaguchi, Masato Takahashi, Yasuo Hozumi, Tomomi Fujisawa, Shozo Ohsumi, Hiromitsu Akabane, Reiki Nishimura, Tsutomu Takashima, Youngjin Park, Yasuaki Sagara, Tatsuya Toyama, Shigeru Imoto, Toshiro Mizuno, Satoshi Yamashita, Satoshi Fujii, Yukari Uemura

**Affiliations:** 1 National Cancer Center Hospital East, Kashiwa, Chiba 277-8577 Japan; 2 Musashino Red Cross Hospital, Musashino, Tokyo 180-0023 Japan; 3 National Hospital Organization Hokkaido Cancer Center, Sapporo, Hokkaido 003-0804 Japan; 4 University of Tsukuba Hospital, Tsukuba, Ibaraki 305-8576 Japan; 5 Gunma Prefectural Cancer Center, Ota, Gunma 373-0828 Japan; 6 National Hospital Organization Shikoku Cancer Center, Matsuyama, Ehime 791-0245 Japan; 7 Asahikawa-Kosei General Hospital, Hokkaido, Asahikawa Japan; 8 Kumamoto Shinto General Hospital, Chuo Ward, Kumamoto 862-8655 Japan; 9 Osaka City University Graduate School of Medicine, Sumiyoshi Ward, Osaka 558-0022 Japan; 10 Tohoku Medical and Pharmaceutical University Hospital, Sendai, Miyagi 981-8558 Japan; 11 Hakuaikai Medical Corp Sagara Hospital, Kagoshima, Japan; 12 Nagoya City University Graduate School of Medical Sciences, Aichi, Nagoya 467-8601 Japan; 13 Kyorin University Hospital, Mitaka, Tokyo 181-8611 Japan; 14 Mie University Hospital, Tsu, Mie 514-8507 Japan; 15 National Cancer Center Research Institute, Chuo-ku, Tokyo 104-0045 Japan; 16 Division of Pathology, Exploratory Oncology Research & Clinical Trial Center, National Cancer Center, Kashiwa, Chiba 277-8577 Japan; 17 National Center for Global Health and Medicine, Tokyo, Japan

**Keywords:** Breast cancer, Breast cancer

## Abstract

**Background:**

The effectiveness of a therapeutic strategy that switches chemotherapy, based on Ki-67 tumour expression after initial therapy, relative to that of standard chemotherapy, has not been evaluated.

**Methods:**

Patients were randomly assigned to the control arm or the Ki-67 response-guided arm (Ki-67 arm). Primary tumour biopsies were obtained before treatment, and after three once-weekly doses of paclitaxel and trastuzumab to assess the interim Ki-67 index. In the control arm, paclitaxel and trastuzumab were continued for a total of 12 doses, regardless of the interim Ki-67 index. In the Ki-67 arm, subsequent treatment was based on the interim Ki-67 index. Ki-67 early responder is defined as the absolute Ki-67 value that was <10%, and the percentage of Ki-67-positive tumour cells was reduced by >30% compared with before treatment. Early Ki-67 responders continued to receive the same treatment, while early Ki-67 non-responders were switched to epirubicin plus cyclophosphamide. The primary endpoint was the pathological complete response (pCR) rate.

**Results:**

A total of 237 patients were randomised. There was almost linear correlation between the Ki-67 reduction rate at interim assessment and the pCR rate. The pCR rate in Ki-67 early non-responders in the Ki-67 arm was inferior to that in the control arm (44.1%; 31.4–56.7; *P* = 0.025).

**Conclusions:**

The standard chemotherapy protocol remains as the recommended strategy for patients with HER2-positive breast cancer.

**Clinical trial registration:**

Clinical Trial Registration: UMIN-CTR as UMIN000007074.

## Background

Estimates suggest that HER2-positive breast cancer accounts for 15–20% of all breast cancer cases, and HER2 is an independent prognostic factor.^1^ Trastuzumab, a humanised monoclonal antibody against HER2, has been reported to markedly improve the prognosis of patients with HER2-positive breast cancer.^2,3^ Accordingly, trastuzumab is widely used as a standard therapy for HER2-positive breast cancer. Although studies suggest that a high pathological complete response (pCR) rate may be achieved by incorporating trastuzumab into preoperative chemotherapy,^4,5^ novel therapeutic strategies are required to increase the pCR rate.

Currently, preoperative and postoperative chemotherapy regimens are almost entirely predetermined by clinical evidence and expert consensus based on the risk factors of recurrence and subtype of breast cancer.^6^ However, tumour tissues are heterogeneous, and the effects of a particular chemotherapeutic regimen are not the same in all patients. Because the standard treatment guidelines are reflective of data from a large sample size, they do not account for individual differences in the response to chemotherapy.

The nuclear proliferation marker Ki-67 has potential utility in the clinical management of breast cancer.^7–9^ Several studies have demonstrated that Ki-67 values after preoperative chemotherapy are indicative of prognosis.^10,11^ Therefore, it may be hypothesised that Ki-67 is suitable as a biological response marker. Accurate assessment of therapeutic responses using a biological marker would enable continuous administration of chemotherapy regimens with poor efficacy to be stopped, and the introduction of different drugs with potentially higher efficacy at an earlier stage. This strategy is considered to be of great relevance in the context of precision medicine.

The aim of this prospective, Phase 2 study was to compare the effectiveness of drug switching, using the Ki-67 index as a biological marker of early responses, with that of the conventional strategy of a continuous pre-specified chemotherapy regimen. Cancer biopsies were obtained during treatment to analyse the biological response.

## Methods

### Patients

This study enrolled women aged 20–75 years with histologically confirmed, invasive HER2-positive breast cancer. HER2 positivity was defined as overexpression by immunohistochemistry (3+) or amplification by fluorescent in situ hybridisation (FISH) according to the 2013 ASCO/CAP Guidelines on HER2 Testing in Breast Cancer. Patients with stage II or III breast cancer, for whom neoadjuvant chemotherapy (NAC) is indicated, were eligible. Other inclusion criteria were Eastern Cooperative Oncology Group performance status (ECOG PS) of 0 or 1, left ventricular ejection fraction (LVEF) ≥ 55% by echocardiography or multigated acquisition (MUGA) scan and preserved organ functions. Exclusion criteria included the presence of synchronous or metachronous malignant cancers, metachronous or bilateral breast cancer, prior or concurrent cardiac disease and uncontrolled hypertension or diabetes mellitus. Written informed consent was obtained from all patients. This trial was registered at UMIN-CTR as UMIN000007074.

### Study design and treatment

This was a multicentre, randomised Phase 2 study. Patients were randomly assigned in a 1:1 ratio to the control arm or the Ki-67 response-guided arm (Ki-67 arm). Randomisation was stratified according to stage (II vs III) and oestrogen or progesterone receptor status (positive vs negative). Before treatment, primary tumour biopsies were obtained from all patients, and Ki-67 levels were assessed.

All patients received initial treatment with paclitaxel (80 mg/m^2^) and trastuzumab (loading dose of 4 mg/kg followed by 2 mg/kg). After three doses of paclitaxel and trastuzumab, the primary tumour was biopsied for interim assessment of the Ki-67 index. Patients assigned to the control arm continued on once-weekly paclitaxel and trastuzumab for a total of 12 additional doses, regardless of the interim Ki-67 index. Patients assigned to the Ki-67 arm received subsequent treatment according to the interim Ki-67 index. When the absolute Ki-67 value was <10% and the percentage of Ki-67-positive tumour cells was reduced by ≥30% compared with before treatment (Ki-67 early responder), once-weekly paclitaxel plus trastuzumab was continued for a total of 12 doses. When the absolute Ki-67 value was ≥10% or the Ki-67 index was reduced by <30% compared with before treatment (Ki-67 early non-responder), chemotherapy was switched to epirubicin (90 mg/m^2^) plus cyclophosphamide (600 mg/m^2^) (EC), administered every 3 weeks for three cycles, combined with once-weekly trastuzumab administered for a total of 12 doses. In cases with an insufficient tumour sample for interim Ki-67 assessment, paclitaxel and trastuzumab were continued. After completion of trastuzumab chemotherapy, patients underwent appropriate surgery according to the size and location of the primary tumour. Recommendations for postoperative treatment included tamoxifen or an aromatase inhibitor for 5 years for hormone receptor-positive tumours, radiotherapy for patients who underwent breast-conserving surgery and had positive axillary lymph nodes pathologically after NAC and trastuzumab for a total of 1 year. The addition of postoperative chemotherapy was permitted.

The primary endpoint was the pCR rate, defined as the complete absence of invasive tumours in breast and axillary lymph nodes. The presence of in situ disease was allowed as pCR. Secondary endpoints included the objective response rate (ORR), disease-free survival (DFS) and overall survival (OS). Adverse events were graded according to the Common Terminology Criteria for Adverse Events (version 4.0). The protocol was reviewed and approved by all the responsible local ethics committees.

### Ki-67 assessment

The primary tumour was biopsied before treatment, and after three doses of once-weekly paclitaxel and trastuzumab. Because a significant level of inter-laboratory and inter-observer variability has been reported, seven, both pretreatment and interim tumour biopsy specimens were centrally processed and evaluated pathologically. At each participating institution, specimens were fixed in 10% neutral buffered formalin and sent to a commercial laboratory (SRL Inc., Tokyo, Japan). Ki-67 was stained immunohistochemically with anti-MIB-1 monoclonal antibodies (Dako Inc., Tokyo, Japan). The Ki-67 index, which was expressed as the percentage of positively stained tumour cells among the total number of tumour cells assessed, was evaluated by an experienced and certificated pathologist (S.F.). The pathologist followed the current recommendations for Ki-67 assessment.^7^ Scoring involved the counting of at least 1000 cells in fields that were identified as representative on an initial overview of the whole section.

### Statistical analysis

Calculations demonstrated that 170 Ki-67 early non-responders were required for a statistical power of 88% to detect a 20% increase in the pCR rate in the Ki-67 arm, assuming a pCR rate of 10% in the control arm and type 1 error of 5%. The expected proportion of Ki-67 early non-responders in the study population was ~60%, and 300 patients were to be enrolled in the study. An interim analysis of the primary endpoint for futility was planned using data from the first 200 patients registered. The chi-squared test was used to analyse differences in the pCR rate between the two arms among the Ki-67 early non-responders. All patients whose Ki-67 index was assessed were included in the efficacy analysis. All analyses were performed using SAS software, version 9.4.

## Results

### Patient population

**Fig. 1:**
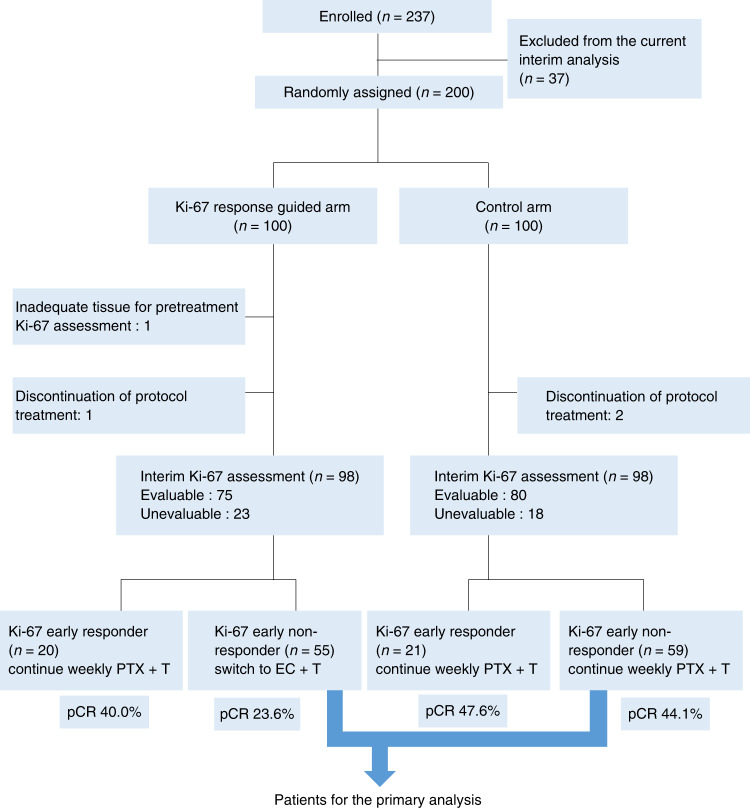
CONSORT diagram for this study. PTX, paclitaxel; EC, epirubicin and cyclophosphamide; T, trastuzumab.

Between December 2011 and September 2015, 237 patients from 12 participating sites in Japan were randomly assigned: 118 to the Ki-67 arm and 119 to the control arm. A pre-specified interim analysis was conducted in August 2015 using data from the first 200 patients. A CONSORT diagram is shown in Fig. 1. At interim assessment, Ki-67 evaluation could not be implemented in 23 cases in the Ki-67 arm and 18 cases in the control arm. In most of these cases, tumour sampling by core-needle biopsy was difficult due to the reduction in tumour size after three doses of trastuzumab plus paclitaxel. The baseline characteristics of patients included in the interim analysis are shown in Table 1. At baseline, the characteristics of the control arm and the Ki-67 arm were well-balanced, including the frequencies of infiltrating duct carcinoma (92%), and ER- (52%) and axillary lymph node-positive patients (≥60%).

**Table 1:** Baseline characteristics of the study population.

			Total (*n* = 200)		Control arm (*n* = 100)		Ki-67 response-guided arm (*n* = 100)		*P* value**
			*n*	%	*n*	%	*n*	%	
Age, years		Median (range)	57.3 (25–75)		56.7 (25–72)		58.3 (31–75)		0.115
Pretreatment Ki-67 index*		Mean (SD)	51.8 (17.8)		49.9 (16.6)		53.6 (18.8)		0.150
Tumour size by palpation, (cm)		Mean (SD)	4.2 (2.8)		3.8 (1.6)		4.5 (3.5)		0.0752
Clinical tumour stage^†^		cT1	16	8.0	11	11.0	5	5.0	0.403
		cT2	145	72.5	70	70.0	75	75.0	
		cT3	26	13.0	12	12.0	14	14.0	
		cT4	10	5.0	6	6.0	4	4.0	
		NA	3	1.5	1	1.0	2	2.0	
Clinical nodal status^†^		cN0	73	36.5	32	32.0	41	41.0	0.124
		cN1	99	49.5	55	55.0	44	44.0	
		cN2	16	8.0	5	5.0	11	11.0	
		cN3	12	6.0	8	8.0	4	4.0	
Clinical stage^†^		I	0	0.0	0	0.0	0	0.0	0.481
		IIA	80	40.0	37	37.0	43	43.0	
		IIB	68	34.0	37	37.0	31	31.0	
		IIIA	32	16.0	14	14.0	18	18.0	
		IIIB	7	3.5	3	3.0	4	4.0	
		IIIC	13	6.5	9	9.0	4	4.0	
Histology		Ductal invasive	185	92.5	92	92.0	93	93.0	0.845
		Lobular invasive	7	3.5	3	3.0	4	4.0	
		Other	8	4.0	5	5.0	3	3.0	
Hormone status^†,‡^	Receptor	ER−/PgR−	91	45.5	45	45.0	46	46.0	1.000
		ER−/PgR+	5	2.5	3	3.0	2	2.0	
		ER+/PgR−	37	18.5	18	18.0	19	19.0	
		ER+/PgR+	67	33.5	34	34.0	33	33.0	

*SD* standard deviation, *NA* not available.

^*^Central assessment.

^†^TNM classification (7th edition).

^‡^Local assessment.

^**^Fisher’s exact test for categorical variables, *T* test for continuous variables.

### Efficacy

**Fig. 2:**
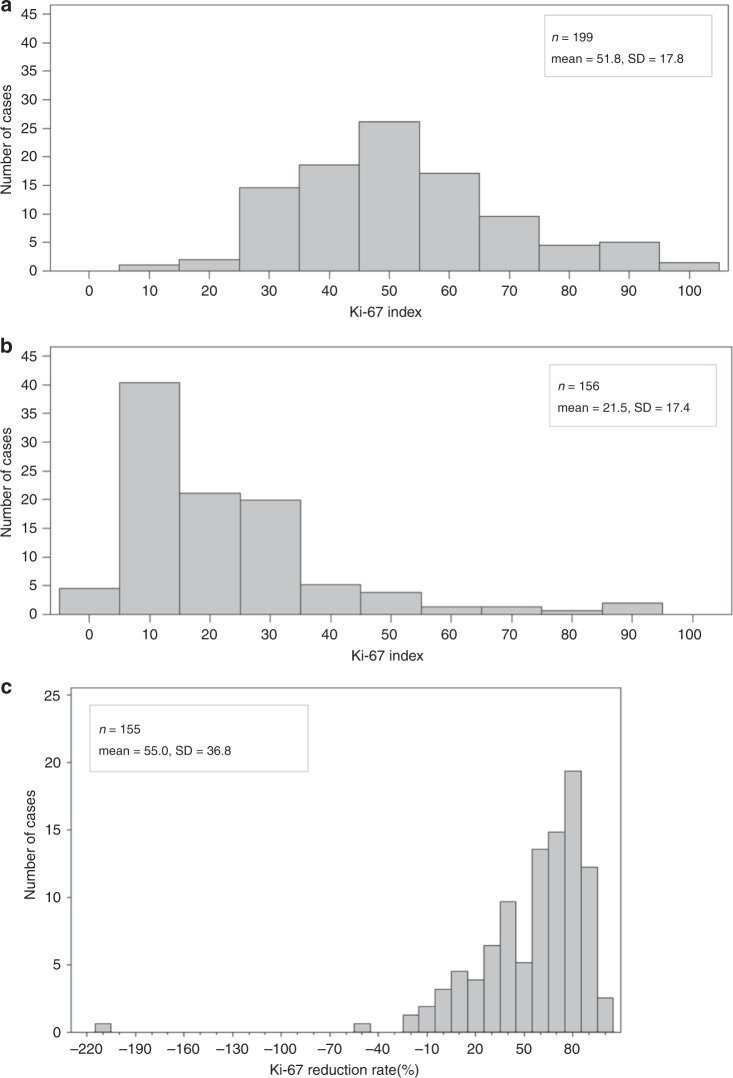
Distribution of the Ki-67 index. At pretreatment (**a**) and interim assessment (**b**). The change in the distribution of the Ki-67 reduction rate from before treatment to interim Ki-67 assessment (**c**). SD, standard deviation.

The distribution of the Ki-67 index before treatment (Fig. 2a) and at interim assessment (Fig. 2b), and the corresponding reductions in the Ki-67 index were analysed (Fig. 2c). Among the 196 evaluable patients in the interim Ki-67 assessment, 114 patients were Ki-67 early non-responders. As shown in Table 2, the pCR rate in Ki-67 early non-responders in the Ki-67 arm (23.6%, 95% CI: 12.4–34.9) was inferior to that of Ki-67 early non-responders in the control arm (44.1%, 95% CI: 31.4–56.7; *P* = 0.025). The Bayesian predictive probability of rejecting the null hypothesis at the end of the study was <1%, which was far below the predefined threshold of 10%. This result met the pre-specified futility criteria of the study protocol. Therefore, the Data Safety Monitoring Committee recommended that patient registration was ceased.

**Table 2:** Pathological complete response rate in each arm among Ki-67 early non-responders and responders.

		Total	pCR		
		*n*	*n*	%	95% CI
Control arm (*n* = 80)	Ki-67 early non-responder*	59	26	44.1	31.4–56.7
	Ki-67 early responder	21	10	47.6	26.3–69.0
Ki-67 response-guided arm (*n* = 75)	Ki-67 early non-responder^†^	55	13	23.6	12.4–34.9
	Ki-67 early responder^‡^	20	8	40.0	18.5–61.5

*pCR* pathological complete response, *CI* confidence interval.

Pathological response was not available in *one patient, ^†^two patients and ^‡^one patient.

**Fig. 3:**
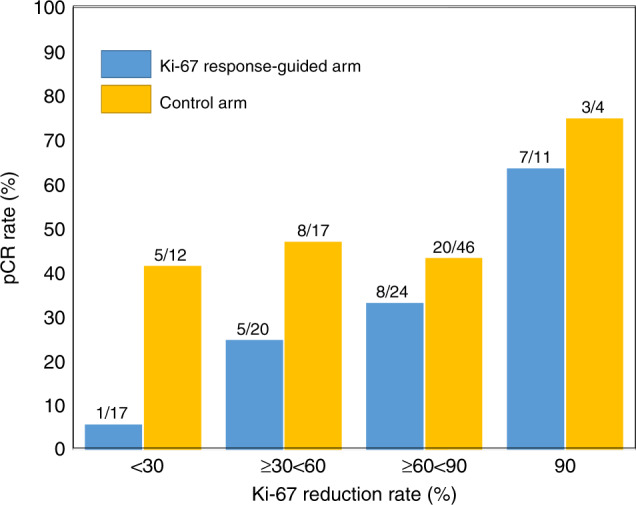
Relationship between Ki-67 reduction rate and pCR rate in each arm. The number above each bar denotes “the number of patients with pCR/all patients”.

In all Ki-67 early non-responders, the interim Ki-67 index was >10% (Supplementary Table 1). Data were analysed from a subgroup of 31 Ki-67 early non-responders, comprising 18 patients from the Ki-67 arm and 13 patients from the control arm, all of whom had an interim Ki-67 index of >10% and a reduction rate of <30% compared with before treatment (Supplementary Table 1). Interim Ki-67 assessment was not possible in 23 cases in the Ki-67 arm and 18 cases in the control arm. The pCR rate of these patients was ~80%. Further analysis was conducted with the inclusion of this subgroup in “the analysis set for efficacy” calculations, assuming that the Ki-67 values at interim assessment were 0. The results are shown in Supplementary Table 2. An almost linear correlation between the Ki-67 reduction rate and the pCR rate was detected (Supplementary Fig. 1). Subgroup analysis according to the Ki-67 reduction rate also produced similar results (Fig. 3).

### Toxicity

Leucopenia, neutropenia, nausea and mucositis were more frequent in the Ki-67 arm, which may be attributed to EC treatment (Supplementary Table 3). Treatment-related adverse events leading to discontinuation of protocol treatment occurred in 3.5% (*n* = 7) of patients (5% in the Ki-67 arm and 2% in the control arm).

### Relationship between Ki-67 reduction and clinical response rates

A strong correlation was detected between the Ki-67 reduction rate and the pCR rate, as well as between tumour size reduction and the pCR rate (Table 3). In contrast, Ki-67 reduction was not related to tumour size reduction rate (Spearman’s correlation coefficient 0.22) (Supplementary Fig. 2). Multivariate analysis demonstrated that Ki-67 and tumour size reduction were independent predictive factors of pCR (Supplementary Table 4).

**Table 3:** Comparison of Ki-67 and tumour size in pCR and non-pCR patients.

	Patients with pCR				Patients with non-pCR				*P* value
	*n*	Mean	SD	(Q1, median, Q3)	*n*	Mean	SD	(Q1, median, Q3)	
Interim Ki-67 index	58	18.8	16.9	(10.0, 19.7, 30.0)	94	22.6	16.8	(5.3, 14.3, 25.6)	0.064^‡^
Ki-67 reduction rate (%)	57	64.6	28.2	(31.2, 59.8, 76.5)	94	49.6	40.3	(47.2, 71.1, 83.3)	0.0058^‡^
Interim tumour size (cm)*	52	2.2	1.3	(1.5, 2.0, 3.0)	80	2.9	1.7	(1.9, 2.5, 3.6)	0.015^†^
Tumour size reduction rate (%)	50	45.1	26.2	(33.3, 40.0, 54.0)	79	30.9	25.3	(12.5, 28.6, 42.9)	0.0027^†^

*pCR* pathological complete response, *SD* standard deviation, *Q1* 1st quartile, *Q3* 3rd quartile.

*Determined by palpation. ^†^Wilcoxon test. ^‡^*T* test.

## Discussion

The pCR rate in the Ki-67 response-guided arm was inferior to that in the control arm. Similar results were obtained after changing the cut-off value for Ki-67 reduction to redefine patients as early non-responders and responders. Also, it was confirmed that the four patients with missing data for pCR do not affect the conclusion, that is, Ki-67 arm was inferior to that in control arm (data not shown).

Therefore, the trial was terminated for futility and patient registration was stopped.

Ki-67 early non-responders were defined by “an absolute Ki-67 value of ≥10% or a reduction in Ki-67 index of <30% after three doses of trastuzumab plus paclitaxel compared with before treatment”. This definition was based on data indicating that a change of at least 21–50% in the Ki-67 reduction rate is needed to provide conclusive evidence of a decrease in Ki-67 due to therapy.^12^ In addition, another report demonstrated that a reduction of at least 25% in Ki-67 after the first course of chemotherapy was associated with a good prognosis.^13^

It was somewhat surprising to observe that switching to EC+ trastuzumab in Ki-67 non-responders led to a lower pCR rate than continuing on paclitaxel + trastuzumab. Taxanes are cell cycle-specific agents, and bind with high affinity to microtubules resulting in inhibition of mitosis and cell death. High Ki-67 expression in tumour cells exhibits rapid-cycling cells. Thus, we speculate that continuing paclitaxel was more effective for high-proliferating tumour cells in Ki-67 non-responders. Indeed, high Ki-67 index was shown to be a potential predictive factor for the benefit of adding taxanes in the adjuvant setting.^14,15^ However, a more convincing rationale remains to be seen. This prospective study was conducted to examine the effectiveness of a therapeutic strategy that switches drugs during treatment, according to the biological tumour response at interim Ki-67 assessment, compared with the existing strategy, in which a continuous predetermined chemotherapy regimen is administered. Some retrospective studies have explored the correlation between Ki-67 and prognosis;^11,16^ however, to the best of the authors’ knowledge, this is the first prospective study to compare the effects of switching drugs based on changes in Ki-67 values. Non-comparative, prospective studies that evaluate drug switching according to clinical responses during treatment have been previously reported.^17–19^ However, in the present study, the correlation between clinical response and the Ki-67 reduction rate was low; therefore, the meanings of those studies and this study are different.

This study has the advantage that it only included patients with HER2-positive cancer, and Ki-67 assessment was performed following the current recommendations for Ki-67 assessment. The results were robust because they were unaltered after redefining Ki-67 early non-responders and responders by changing the cut-off value for the Ki-67 reduction rate. The limitations of this study include ki-67 assessment was performed by a single pathologist, the primary endpoint of pCR rate and the lack of long-term follow-up data. In addition, this was a Phase 2 study with a relatively small sample size. Long-term data will be presented in the future.

Anthracyclines and taxane are not cross-resistant.^20^ They have almost equivalent activity,^21^ and are commonly used as preoperative and postoperative chemotherapy. EC therapy comprises epirubicin, which has lower cardiac toxicity than other anthracyclines when administered in combination with trastuzumab; therefore, EC therapy was administered to Ki-67 early non-responders in this study. The incidence of cardiac toxicity was within the range of that reported in previous studies.^20–23^

Although the results of this study were negative, it is unclear whether this was related to the use of EC in Ki-67 early non-responders, or the drug-switching strategy per se. If the former is the cause, the development of novel anticancer drugs may enhance the prognosis of Ki-67 early non-responders. Taxane seems to work later than the time point for Ki-67 reduction. However, if we set the time point later, the meaning of Ki-67 response-guided arm is limited. We wanted to judge the initial drug effectiveness at an early time point with Ki-67 response because we can discard the ineffective drug earlier. The correlation between the clinical response rate and the reduction in Ki-67 was low; therefore, the clinical relevance of examining Ki-67 levels throughout treatment remains to be clarified.

In conclusion, the effectiveness of a therapeutic strategy that switches chemotherapy, based on Ki-67 tumour expression after initial therapy, relative to that of standard chemotherapy, was evaluated in patients with HER2-positive breast cancer. The pCR rate in the Ki-67 response-guided arm was inferior to that in the control arm. The standard chemotherapy protocol remains as the recommended strategy for patients with HER2-positive breast cancer.

## Supplementary information

supplementary files

## Data Availability

The data generated and analysed during this study are available at UMIN-CTR as UMIN000007074.
