# Supplementary material for: Ki-67 response-guided preoperative chemotherapy for HER2-positive breast cancer: results of a randomised Phase 2 study
Source: Br J Cancer. 2020 Apr 2;122(12):1747–53. doi: 10.1038/s41416-020-0815-9 (PMC7283228; doi:10.1038/s41416-020-0815-9)
Supplement: Supplementary file 1 — supplementary files [file 41416_2020_815_MOESM1_ESM.docx]

Supplementary Figure 1.


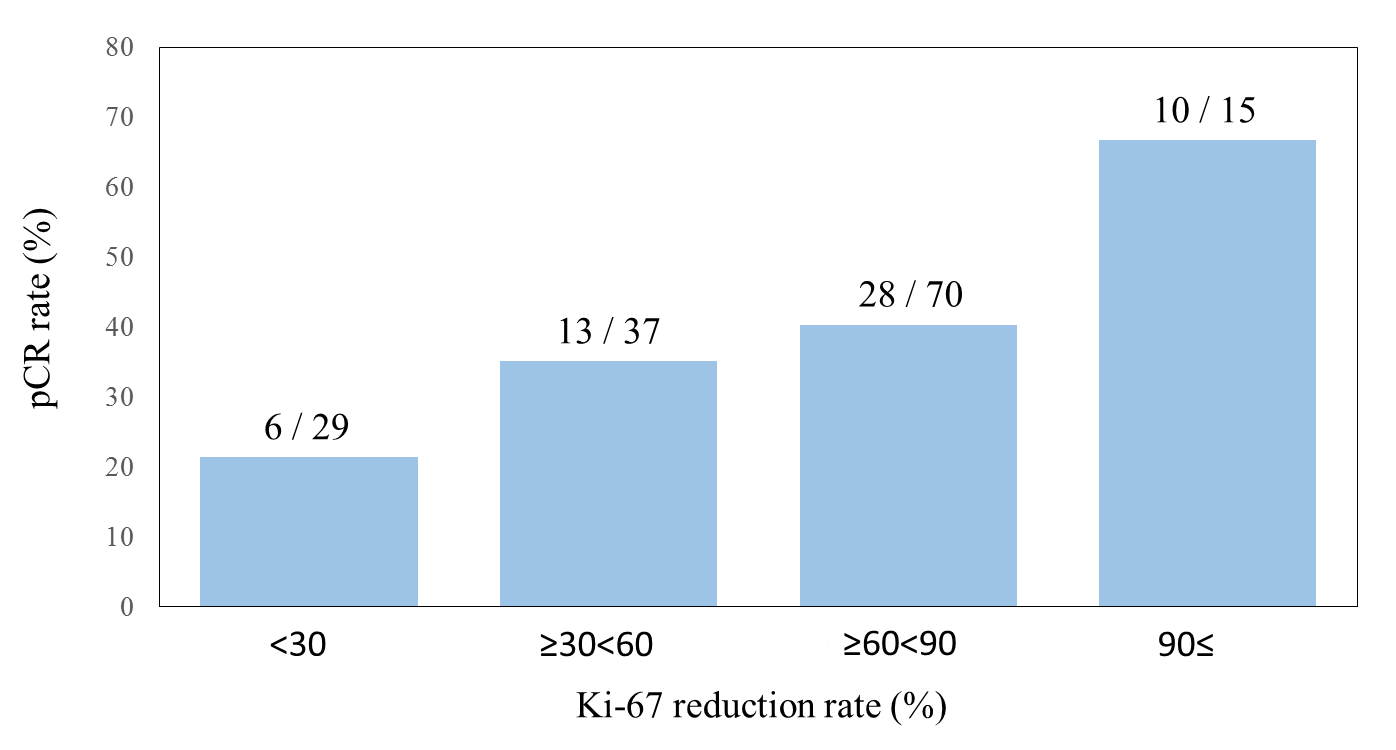


Supplementary Figure 2.


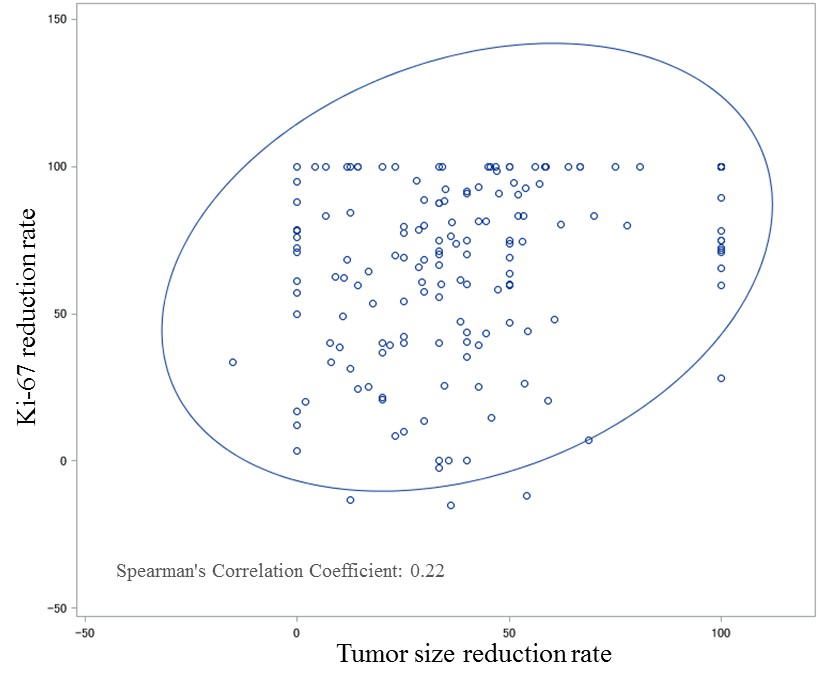


Supplementary Table 1. Details of Ki-67 responses at interim Ki-67 assessment

|  | Interim Ki-67 index (n) | | |
| --- | --- | --- | --- |
|  | | ≥10% | <10% |
| Ki-67 reduction rate (n) | |  |  |
| <30% | | 31 | 0 |
| ≥30% | | 83 | 41 |

Supplementary Table 2. Pathological complete response rate in each arm among Ki-67 early non-responders and responders

|  |  | Total | pCR | | |
| --- | --- | --- | --- | --- | --- |
|  |  | n | n | % | 95%CI |
| Ki-67 early non-responder (n=114) | Control arm* | 59 | 26 | 44.1 | 31.4 - 56.7 |
|  | Ki-67 response-guided arm† | 55 | 13 | 23.6 | 12.4 – 34.9 |
| Ki-67 early responder  (n=82) | Control arm‡ | 39 | 24 | 61.5 | 46.3 - 76.8 |
|  | Ki-67 response-guided arm§ | 43 | 28 | 65.1 | 50.9 - 79.4 |

Abbreviations: pCR, pathological complete response; CI, confidence interval.

Pathological response was not available in *1 patient, †2 patients, ‡1 patient and §1 patient.

Supplementary Table 3. Adverse events

|  | Control arm (n=100) | | | |  | Ki-67 response-guided arm (n=100) | | | |
| --- | --- | --- | --- | --- | --- | --- | --- | --- | --- |
|  | Grade1 | Grade2 | Grade3 | Grade4 |  | Grade1 | Grade2 | Grade3 | Grade4 |
| Hematological (n) |  |  |  |  |  |  |  |  |  |
| Leucopenia | 20 | 18 | 5 | 0 |  | 14 | 27 | 13 | 6 |
| Neutropenia | 7 | 17 | 7 | 1 |  | 9 | 20 | 9 | 14 |
| Anemia | 31 | 6 | 0 | 0 |  | 32 | 20 | 3 | 0 |
| Thrombocytopenia | 2 | 0 | 0 | 0 |  | 5 | 1 | 0 | 0 |
| Increased AST | 0 | 7 | 0 | 0 |  | 0 | 3 | 1 | 0 |
| Increased ALT | 0 | 6 | 3 | 0 |  | 0 | 3 | 6 | 0 |
| Hyperglycemia | 0 | 0 | 1 | 0 |  | 0 | 0 | 0 | 0 |
| Hypoalbuminemia | 0 | 0 | 0 | 0 |  | 0 | 1 | 0 | 0 |
| Non-hematological (n) |  |  |  |  |  |  |  |  |  |
| Nausea | 19 | 4 | 0 | 0 |  | 33 | 10 | 3 | 0 |
| Vomiting | 3 | 3 | 0 | 0 |  | 6 | 1 | 1 | 0 |
| Fatigue | 34 | 6 | 2 | 0 |  | 34 | 10 | 4 | 0 |
| Mucositis | 16 | 3 | 0 | 0 |  | 21 | 9 | 3 | 0 |
| Arthralgia | 24 | 2 | 0 | 0 |  | 23 | 1 | 0 | 0 |
| Myalgia | 18 | 1 | 0 | 0 |  | 16 | 0 | 0 | 0 |
| Diarrhea | 13 | 3 | 0 | 0 |  | 13 | 1 | 0 | 0 |
| Peripheral neuropathy |  |  |  |  |  |  |  |  |  |
| sensory | 60 | 17 | 0 | 0 |  | 45 | 11 | 2 | 0 |
| motor | 1 | 1 | 0 | 0 |  | 1 | 2 | 0 | 0 |
| Anorexia | 0 | 0 | 0 | 0 |  | 0 | 2 | 0 | 1 |
| Infection | 0 | 1 | 4 | 0 |  | 0 | 2 | 0 | 0 |
| Rash | 0 | 4 | 0 | 0 |  | 0 | 0 | 0 | 0 |
| Nail loss | 2 | 1 | 0 | 0 |  | 1 | 0 | 0 | 0 |
| Arrhythmia | 0 | 0 | 0 | 0 |  | 1 | 0 | 0 | 0 |
| Heart failure | 0 | 0 | 0 | 0 |  | 1 | 0 | 0 | 0 |
| Abdominal pain | 0 | 1 | 0 | 0 |  | 0 | 0 | 0 | 0 |
| Edema | 0 | 1 | 0 | 0 |  | 0 | 1 | 0 | 0 |
| Dry eye | 0 | 1 | 0 | 0 |  | 0 | 0 | 0 | 0 |
| Dyspepsia | 0 | 1 | 0 | 0 |  | 0 | 0 | 0 | 0 |
| Dental caries | 0 | 0 | 0 | 0 |  | 0 | 1 | 0 | 0 |
| Headache | 0 | 1 | 0 | 0 |  | 0 | 1 | 0 | 0 |
| Infusion related reaction | 0 | 1 | 0 | 0 |  | 0 | 1 | 0 | 0 |
| Paronychia | 0 | 0 | 1 | 0 |  | 0 | 0 | 0 | 0 |
| Skin disorders | 0 | 0 | 1 | 0 |  | 0 | 0 | 0 | 0 |
| Taste alterations | 0 | 0 | 0 | 0 |  | 0 | 1 | 0 | 0 |
| Watering eyes | 0 | 0 | 0 | 0 |  | 0 | 1 | 0 | 0 |
| Weight loss | 0 | 0 | 0 | 0 |  | 0 | 1 | 0 | 0 |

Supplementary Table 4. Predictive factors of pCR

| Variable |  |  | OR |  | 95%CI |  | p value‡ |
| --- | --- | --- | --- | --- | --- | --- | --- |
| Tumor baseline* |  |  | 0.84 |  | 0.64 - 1.11 |  | 0.22 |
| Ki-67 baseline* |  |  | 1.01 |  | 0.98 - 1.03 |  | 0.60 |
| Tumor reduction† | 10% |  | 1.18 |  | 1.01 - 1.39 |  | 0.042 |
| Ki-67 reduction† | 10% |  | 1.17 |  | 0.99 - 1.38 |  | 0.066 |
| Estrogen receptor | positive |  | 1 |  |  |  |  |
|  | negative |  | 3.45 |  | 1.21- 9.86 |  | 0.021 |
| Progesterone receptor | Positive |  | 1 |  |  |  |  |
|  | negative |  | 2.28 |  | 0.75 – 6.94 |  | 0.15 |
| Clinical node status | cN0 |  | 1 |  |  |  |  |
|  | cN1 |  | 1.87 |  | 0.36 – 9.55 |  | 0.45 |
|  | cN2/cN3 |  | 1.60 |  | 0.14 – 18.02 |  | 0.70 |
| Clinical stage | ⅡA |  | 1 |  |  |  |  |
|  | ⅡB |  | 1.12 |  | 0.23 – 5.58 |  | 0.89 |
|  | ⅢA/ⅢB/ⅢC |  | 1.27 |  | 0.14 – 11.26 |  | 0.83 |

Abbreviation: pCR, pathological complete response; OR, Odds Ratio; CI, confidence interval.

* Included in the model as continuous variable. The estimated OR represents the average increase of pCR per 1cm increase

† Included in the model as continuous variable The estimated OR represents the average increase of pCR per 10% increase

‡P value estimated by logistic regression model

Figure legend

Supplementary Fig. 1 The relationship between Ki-67 reduction rate and pCR rate. The number above each bar denotes ‘the number of patients with pCR / all patients’.

Supplementary Fig. 2 A correlation plot of the Ki-67 reduction rate and tumor size at interim assessment. One case with a reduction rate of -220% was removed from the data shown in the figure.

List of the names of the ethics committees approved this study

・Institutional Review Board of National Cancer Center Hospital

・Institutional Review Board of Musashino Red Cross Hospital

・Institutional Review Board of National Hospital Organization Hokkaido Cancer Center

・Institutional Review Board of University of Tsukuba Hospital

・Institutional Review Board of Gunma Prefectural Cancer Center

・Institutional Review Board of National Hospital Organization Shikoku Cancer Center

・Institutional Review Board of Asahikawa-Kosei General Hospital

・Institutional Review Board of Kumamoto Shinto General Hospital

・Institutional Review Board of Osaka City University Graduate School of Medicine

・Institutional Review Board of Tohoku Medical and Pharmaceutical University Hospital

・Institutional Review Board of Hakuaikai Medical Corp Sagara Hospital

・Institutional Review Board of Nagoya City University Graduate School of Medical Sciences

・Institutional Review Board of Kyorin University Hospital

・Institutional Review Board of Mie University Hospital
